# Thermal Face Verification through Identification

**DOI:** 10.3390/s21093301

**Published:** 2021-05-10

**Authors:** Artur Grudzień, Marcin Kowalski, Norbert Pałka

**Affiliations:** Institute of Optoelectronics, Military University of Technology, 2 Gen. S. Kaliskiego St., 00-908 Warsaw, Poland; norbert.palka@wat.edu.pl

**Keywords:** face verification, long-wavelength infrared radiation, convolutional neural networks

## Abstract

This paper reports on a new approach to face verification in long-wavelength infrared radiation. Two face images were combined into one double image, which was then used as an input for a classification based on neural networks. For testing, we exploited two external and one homemade thermal face databases acquired in various variants. The method is reported to achieve a true acceptance rate of about 83%. We proved that the proposed method outperforms other studied baseline methods by about 20 percentage points. We also analyzed the issue of extending the performance of algorithms. We believe that the proposed double image method can also be applied to other spectral ranges and modalities different than the face.

## 1. Introduction

To date, most of the research on face recognition has focused on the visible range of radiation [1]. Recently, a growing interest has been observed in face recognition by the long-wavelength infrared radiation (LWIR) ranging from 8 to 12 μm [2]. Thermal cameras are passive devices which can work in low light conditions and are highly resistant to illumination changes [3]. Registered radiation is proportional to the relative distribution of the apparent temperature of objects placed in the field of the camera view and their emissivity. In addition, thermal cameras offer spoofing detection capability [4].

Face recognition in the thermal infrared spectrum has not been explored extensively. The main reason for the lower popularity in the thermal infrared domain is the higher cost of cameras and a still relatively small number of scientific image datasets. Visible light cameras are more accessible with prices starting from tens of dollars compared to the thermal infrared sensors that cost at least several thousand dollars. However, thermal infrared domain has many security and military applications; thus, the exploration of thermal face recognition is highly desired. 

The paper presents a new approach for face verification based on a combination of traditional Siamese architecture with a classification task. The face verification method proposed in this paper was inspired by the Siamese architecture where two images were introduced as an input. Before the feature extraction stage, we combined two face images into one double image consisting of both sample images framed side by side. However, unlike the Siamese architecture, where both images are processed in separate paths, here, the double image is processed in a single path which is similar to the identification with CNNs. The proposed method uses a two-class classification to compute the face verification score. Taking the above under consideration, we call this method the verification through identification (VTI) method. 

There is a very limited number of works related to thermal face recognition; the method presented in this paper was implemented for comparing thermal infrared images. However, it is possible to use the method for other spectral domains as well. A thorough description of the method is provided together with the experimental methodology and results. For reference, results obtained with baseline methods are presented. 

The contributions are summarized as follows:

- A new approach for face verification called verification through identification;
- The use of VTI for thermal face verification;
- Evaluation of existing state-of-the-art methods for face verification in the thermal infrared domain.

The organization of the paper is as follows. Section 2 presents a review of related works. Section 3 consists of a description of three databases used in the presented analysis and the baseline methods used for comparison. Verification through identification is detailed in Section 4. VTI performance is compared with the baseline methods in Section 5. Finally, the work is summarized, and further applications of the developed method are presented.

## 2. Related Works

Since there is a limited number of works on face recognition in the thermal infrared domain, we first focus on analyzing existing methods in the visible spectrum. Face recognition in the visible range can be based on appearance (3D morphable models [5], 2D active appearance model [6], and elastic bunch graph matching method [7]) or texture (local feature descriptors, such as scale invariant feature transform [8], local binary pattern (LBP) [9], histogram of oriented gradients (HOG) [10], and local derivative patterns (LDP) [11] with statistical decision functions, such as support vector machines (SVMs) [12], linear discriminant analysis [13], principle component analysis [14], independent component analysis [15], or convolutional neural networks (CNNs) [16]). 

Two-dimensional face recognition in visible light often struggles with non-uniform and varying illumination. A considerable part of face recognition methods uses 3D data to avoid illumination issues. As an example, Mantecón et al. proposed novel depth face descriptor called bag of dense derivative depth patterns (Bag-D3P), which more precisely extracts specific characteristics of depth images, and exploits the extended spatial and depth resolutions of the latest 3D cameras [17]. You et al. proposed a multi-channel deep 3D face network for face recognition based on 3D face data that uses nine channels instead of three in the input layer [18]. Pini et al. [19] used VGG16, ResNet-18 and InceptionV3 without the last classification layer for the extraction of features from depth images. Authors compared probe and gallery depth maps, computing the cosine similarity between the deep features that were extracted by the networks. For every probe, they selected the predicted identity as the gallery candidate corresponding to the maximum similarity. The paper also introduced a new database composed of several facial modalities, including thermal infrared face images. 

Several approaches for facial verification have been proposed, including Siamese, Triplet and Quadruplet architectures. The Siamese network consists of two identical neural networks that process two images in parallel paths. The two processing paths are connected at the end and fed the last layer which calculates the similarity between the two images. In the method introduced by Chopra et al. [20], they selected pairs of images from the same and different subjects, performed feature extraction using neural networks, and then calculated the similarity between the pairs using similarity metric and loss functions. The loss function was designed so as to decrease the energy function of genuine pairs (features derived from the same subjects) and increase the energy of impostor pairs (features derived from different subjects). The energy function was defined as a compatibility measure between two images. The Siamese architecture uses a dedicated feature extraction method for both processing channels and a decision function comparing the samples. Both local descriptors and neural networks can be used as feature extraction methods. 

Regarding local descriptors, Lu et al. proposed to verify both the identity and the kinship between subjects using a pair of images. For each image, they extracted four feature descriptors: LBP, dense scale invariant feature transform, HOG, and local phase quantization. They developed a decision function called discriminative deep multi-metric learning. This decision function learned multiple neural networks so that common and discriminative information could be extracted to make all features more reliable [21]. One of the results presented in their work shows that the feature extraction stage can be modified to obtain better accuracy. They showed this by comparing the results of accuracy for one feature extraction method and for multi-extracted features. For example, accuracy with the LBP method was 81.26%. While many extracted features gave an accuracy of 82.54% for the YTF database.

Schroff et al. presented the Siamese architecture based on the modification of the Face-Resnet architecture for feature extraction [22]. Authors proposed an additional L2-normalization and the addition of a scale layer after a fully connected layer to improve the true acceptance rate (TAR). They showed that the modification of CNN allows a TAR of 0.975 for FAR = 0.1 for the IJB-A database to be achieved. The initial value of TAR was 0.957 [23]. 

The selection of the decision function is the second key factor in the Siamese architecture. Shnain et al. implemented a similarity measure between images for face recognition [24]. They proposed an efficient similarity index that resolved the shortcomings of the existing measures of feature and structural similarity. They combined the best features of the well-known structural similarity index measure (SSIM) and the feature similarity index measure (FSIM). This work showed that proposed feature-based structural measure (FSM) outperforms the conventional SSIM and FSIM in its ability to detect the similarity among similar faces, even under significantly noisy conditions. The FSM produces maximal similarity when the images are similar, while giving near-zero similarity when the images are dissimilar.

Triplet architecture can be considered as a natural progress of the Siamese architecture. It was proved that a higher performance of face verification can be achieved by adding an additional sample at the initial stage of the algorithm which also determines the change in the decision function. The Triplet architecture with three biometric samples, positive, negative, and randomly selected, called an anchor, was introduced for a better separation of impostor and genuine samples by the decision function. Features were extracted from each sample, and then sets of data were learned to meet the assumption that the distance function between the positive examples is reduced and the distance function between the negative examples is increased [22]. The method based on the Triplet architecture achieved an accuracy of 99.63% for the LFW database, while the reference Siamese architecture achieved an accuracy of 97.35% [25].

A similar idea was investigated by Yu et al., which resulted in a development of the Quadruplet architecture based on four images [26]. Two images formed a pattern and a test image, while two others were derived from two other randomly selected subjects. In this case, the loss function consisted of two margin parameters: the distance between positive and negative pairs containing the same face image and the distance between positive and negative pairs containing different face images. The presented Quadruplet method achieved Rank-1 of 48.42% compared with 28.16% achieved by the Triplet architecture.

As far as face identification in LWIR is concerned, Bi et al. focused on the multi-feature fusion technique. They used four methods of feature extraction: LBP, Gabor jet descriptor, Weber local descriptor, and a down sampling feature [27]. The decision function responsible algorithm was the sparse representation classifier. They minimized the total prediction error of all testing faces and used least-squares and regularization methods for optimal learning to obtain a weight vector. Multi-feature fusion was highlighted by the weight vector computation and the final residual computation components. The method achieved an accuracy of 91.5% for the face identification task using the IRIS thermal face database. 

The work of Rodriguez-Pulecio et al. considered thermal face identification [2]. They implemented thermal signature templates with natural scene statistic features to optimize the model based on the quality-aware complex wavelet similarity index. Their decision function was based on the similarity measure using the Euclidean distance function and threshold to determine whether the subject identity in the test image was recognized. The proposed method uses aligned images which were annotated manually due to the lack of automatic landmark detection method for thermal images. They achieved 86.6% in Rank-1 for the UND-X1 database.

From the above analysis, it can be concluded that the verification performance progress can be achieved by modifying the subsequent steps: architecture inputs (one or two more samples), feature extraction methods (CNNs or local descriptors), and decision functions (similarity measure or metric learning). 

## 3. Methodology

This section provides a description of three databases used for comparative tests. Moreover, two feature extraction methods and two decision functions, which constitute baseline methods, are also described. 

### 3.1. Dataset

**Figure 1:**
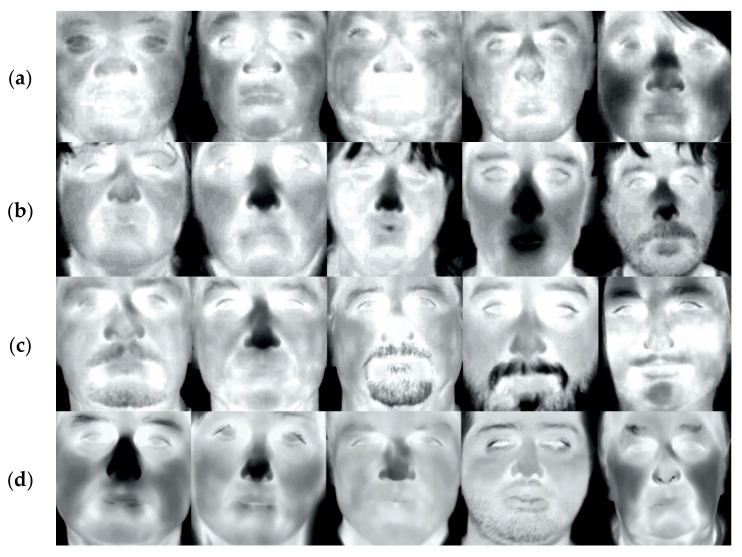
Gallery of thermal face images from (**a**) PROTECT dataset, (**b**) CARL dataset, (**c**) In-House (FLIR A65), (**d**) and In-House (FLIR P640).

The database prepared for the research consisted of 132 subjects selected from three databases: In-House, CARL [28], and PROTECT [29]. The characteristics of each database are presented below. Twelve images were selected per subject. Examples of images from each database are shown in Figure 1.

#### 3.1.1. In-House Database

The In-House dataset collected on the premises of Military University of Technology, Warsaw, Poland, contains images of 44 subjects which were recorded using two thermal infrared cameras, namely, FLIR A65 (resolution 640 × 480, 16 subjects) and FLIR P640 (resolution 640 × 480, 28 subjects). The protocol of the dataset has been partially described in [30]. The dataset was recorded during two sessions, initial and repeated after a year. Since the acquisition process was long, we used two cameras and obtained a database of a wide variety of subjects and variants. Twelve images per subject were acquired—four images with the head turned in four directions and 8 images with the face in a frontal position. The subjects age ranged from 19 to 85 years. The distance between the cameras and the subject was about 1.5 m.

#### 3.1.2. CARL Database

Images were recorded by a TESTO 880-3 camera in the spectral range from 8 to 14 μm. A thermal camera equipped with an uncooled detector with a germanium lens recorded images with a resolution of 160 × 120 pixels. The distance between the user’s face and the camera was 1.35 m. The CARL database contained images of 41 subjects. There were 4 sessions. Fifteen images per subject were recorded for one session. For further research, we selected 12 images for each subject from the first session.

#### 3.1.3. PROTECT Multimodal DB

The database was developed as a part of the Horizon 2020 PROTECT project and contained data on several biometrics modes, including thermal facial images. Thermal images were recorded by FLIR A65 working in the range of 7.7 to 11.5 μm with a resolution of 640 × 512 pixels. The distance between the camera and subject was 1.5 m. The thermal face dataset included 47 subjects with a 57%/43% male/female distribution, aged from 20 to 80 years. Twelve images per subject were recorded—8 images with the face in a frontal position to the camera and four images with the head turned in four directions: left, right, up, and down.

### 3.2. Baseline Methods

For reference purposes, the VTI method was compared with other baseline methods. Due to the limited number of works for thermal face verification, we selected the most popular methods for face verification operating well in the visible domain as the baseline. Images can be pre-processed before the feature extraction. Pre-processing can include filtering, normalization, and face detection. In this work, images were sent to the face detection algorithm based on Faster R-CNN [31,32]. Face images were cropped to 300 × 300 pixels.

#### 3.2.1. Feature Extraction Methods

The feature extraction stage aims to extract distinctive biometric features from each image separately. In this work, we used two methods, local descriptors and neural networks, for feature extraction. The selected set of local descriptors includes LBP, HOG, and LDP. Parameters of the local descriptor algorithms are presented in Table 1.

Sample features may be extracted from an image using certain layers of trained convolutional neural networks. In this case, the set of architectures included AlexNet [33], DenseNet-201 [34], Inception-v1 (GoogLeNet), Inception-v3 [35], InceptionResNet-v2 [36], ResNet-18, ResNet-50, ResNet-101 [37], VGG-16, and VGG-19 [38]. All the images were resized to fit the size of an input layer. The same principle was applied to image layers—if an image did not consist of 3 layers, the layers were duplicated to match the neural network requirements.

For each testing and training set, the feature extraction stage was applied. Features extracted from testing and training sets were paired and categorized into genuine and impostor. Extracted features from testing and training sets were paired with the appropriate labels, 1 and 0 for the same and different subjects, respectively.

#### 3.2.2. Decision Functions

Two feature extraction methods, namely, the convolutional neural network and the local descriptors, as well as two decision functions (metrics and support vector machines) were selected as the baseline methods. Training sets of features extracted both using CNNs and local descriptor algorithms were combined with SVM and metrics. Finally, we received four reference methods, including local descriptors with metrics, local descriptors with SVM, CNNs with metrics, and CNNs with SVM. 

A support vector machine is a multi-classification algorithm which finds an optimal linear or polynomial decision surface [12]. For the purpose of face verification, SVM can be used as a binary classification algorithm. The weighted combination of support vectors for the training set creates a decision surface. Support vectors define a boundary between the two classes only when two sets are analyzed. 

Before feeding the data into the algorithm, the feature vectors were combined into a single vector. If the first feature vector had a dimension of 1 × L1 and the second vector had a dimension of 1 × L2, then the finally obtained vector had a dimension of 1 × (L1 + L2). This produced training samples that should be labeled as 1 when the vectors are from the same person and 0 when they are derived from different subjects.

The second method used in this paper was the decision function-based method which aims to measure similarity using metrics and threshold function. Features extracted from CNNs or local descriptors were paired to obtain pairs of vectors from the same person and from different subjects. The distance functions or metrics provided similarity between two vectors for each pair. We used four distance functions: correlation, Euclidean, cosine, and Spearman. As each distance provides different results, we investigated a variety of configurations and provided the best achieved results. 

The similarity result for similar pairs is described as *D(x, y)* and for dissimilar pairs as *D(x’, y’)*. The similarity *D* of the genuine pair is expected to be small, and the similarity *D* of the impostor pair is expected to be large, so the model needs to meet the following condition:

(1) $$D\left(x,y\right)+m<D\left(x\prime ,y\prime \right)$$

Here, *m* is the margin which allows us to find the decision function threshold for which the following formula is fulfilled:

(2) $$Y\left(x,y\right)=\left\{\begin{array}{c} 1,\;if\;D\left(x,y\right)<th\\ 0,\;otherwise \end{array}\right\}$$

where *Y* is the score of similarity for two feature vectors, *x* and *y*; *th* is the threshold value; and *D* is the distance function for the testing pair [16].

## 4. Verification through Identification

In this paper, we present the neural network-based VTI method which connects some aspects of verification and identification. Although the method provides a result of a classification of two images, it can be considered as a novel approach to face verification. The main difference between the presented approach and the existing methods is the input data structure. In the Siamese and triplet methods, two or three separate images are processed in two or three separate paths, while in the case of VTI, only one image composed of two images is analyzed. Images during processing in VTI, when composed in one image, have only one processing path. 

The data preparation process is as follows. Considering a given set of N images that were split into two equal parts to obtain two temporary sets, pairs of images taken from the two temporary sets are generated. The number of possible combinations includes both pairs of the same and different subjects. Therefore, two conditions must be established. The first condition checks the ID numbers of the generated image pairs. If the ID numbers are equal, the pairs of images are assigned to the genuine class; otherwise, they assigned to the impostor class. In the case of images presenting the same subjects, if possible, component images are extracted from two different recording sessions. In the case of the In-House database component, images were recorded with 1-year intervals. After selecting pairs of images for appropriate classes, we obtain two classes with a different number of pairs of images. The impostor class contains many more pairs than the genuine class. Therefore, the second condition is to randomly select pairs from the genuine class so that the number of pairs in each class is equal. It is important that the data learning algorithm does not assign learning weights to one class.

**Figure 2:**
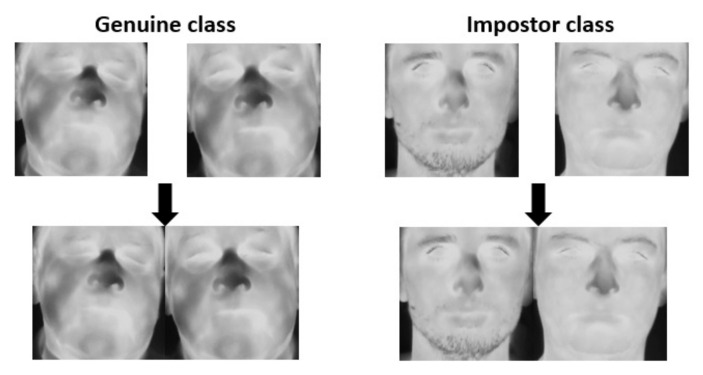
Scheme of combining two images into genuine and impostor classes.

In the next step, pairs of images are processed so that two images compose one image. If a face image has dimensions of *h* × *w*, where *h* is the height and *w* is the width, then a new image is a combination of two images with respect to the edges of their height. Hence, one can define a symbolic vertical line halfway across the new image width which divides the image into two face images (Figure 2).

The two-class dataset can be interpreted and analyzed as a two-class identification approach. Further steps in the VTI method, such as feature extraction and data classification, were based on state-of-the-art CNNs and a SoftMax layer, the same as in a typical classification task. Therefore, the proposed method is called verification through identification, as the purpose is to verify the identity based on a task classification. 

**Figure 3:**
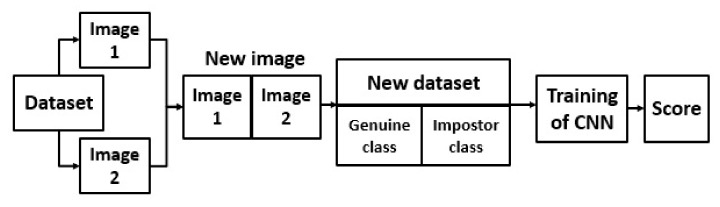
Scheme of the verification through identification method.

For the preparation of training data, we used neural networks which had been pretrained in the ImageNet database, in the same way as the feature extractions tested for baseline methods. The newly prepared images were changed depending on the input layer of the neural network used. This is very important because combining two images into one requires increasing dimensions. The fully connected layer of the neural network model was replaced with a new fully connected layer with two outputs. The scheme of the VTI method is shown in Figure 3.

Each neural network model during training process was optimized using the stochastic gradient descent with momentum [39]. To increase the amount of training data, we also introduced a mirror image augmentation. The learning of the neural network finished when the accuracy given for the validation set no longer improved. In other words, if the validation set accuracy after 20 consecutive runs was not higher than or equal to the previous highest accuracy, then the learning was stopped. The learning speed was related to the learning rate, which was 0.001, and the batch size was 64. The method was implemented and tested in the MATLAB environment.

## 5. Results and Discussion

The method was validated in a 5-fold cross validation scheme. Each section contained a set of test and training data split by 30%/70%. Due to this approach, the results were not affected by the error related to repeating the same image in the training and testing sets, which increased the credibility of the experiments. At the feature extraction stage, the same amount of data was obtained, regardless of the method used. We received 6624 and 2880 images for training and testing datasets, respectively. 

All the results are presented using the true acceptance rate (TAR) and the false acceptance rate (FAR) as the common biometric performance indicators. For each method, the results of TAR obtained for the 5-fold division are presented as the mean with the standard deviation. In this work, we considered many methods and configurations which led to many results and, hence, only the best ones are presented.

### 5.1. Baseline Methods

As for the feature extraction, the input data size for local descriptor methods was of 300 × 300 pixels, while for the neural networks, it depended on the first layer of the selected network. Due to the lack of large face databases in LWIR, the neural network models used for feature extraction were pre-trained in the public ImageNet database of various objects collected in the visible range.

The calculation of the results based on metrics was performed in the same way for each dataset. First, for the training data, the values of the metrics for the respective distance function were determined between the feature vectors. For the received values, a threshold for FAR equal to 1% and 0.1% was calculated. The threshold values from the training data could be used to calculate the TAR value for the testing data.

In Table 2, the percentage share of each database in each dataset from 1 to 5 is presented. Later in the work, we also present the results of TAR for each database separately. In this article, the database names are used in the form of abbreviations: IOEA—In-House dataset acquired with FLIR A65; IOEP—In-House dataset acquired with FLIR P640; CARL—CARL dataset; PROTECT—PROTECT dataset.

For the decision function based on metrics, four distance functions were tested. Table 3 presents the best results for the decision function based on metrics with local descriptors and models of neural networks for four distance functions.

The best result for local descriptor algorithms was obtained for the LBP with the Spearman distance. The TAR value of around 40% for FAR = 0.1% could probably be improved by a better selection of the feature extraction method parameters. For FAR = 0.1%, AlexNet is the best CNN model for the feature extraction, while models with the inception modules reach the lowest values.

The next two methods consisted of decision functions based on SVM with feature extractions using local descriptors and CNNs, respectively. The received scores determined how far the test sample was from the learned function. To present the results, the scores were transformed into posterior probabilities by means of the sigmoid or step function. Table 4 presents the results of the extraction methods with local feature descriptors and neural networks.

The results obtained for local descriptor and CNN models with SVM are insufficiently low, which suggests that these methods cannot be used for the efficient face verification in LWIR. 

**Figure 4:**
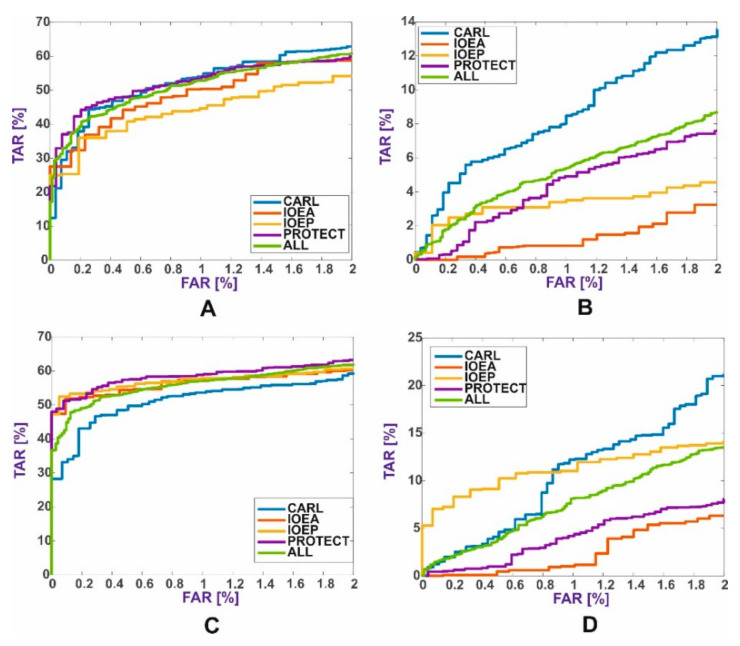
ROC curves for individual databases: (**A**) CNNs with metrics, (**B**) CNNs with SVM, (**C**) local descriptor methods with metrics, and (**D**) local descriptor methods with SVM.

Figure 4 presents the receiver operating characteristic (ROC) curves for each method with the best parameters, broken down into individual databases and the average for all databases.

Given the best method, i.e., the decision function based on metrics, there is no reason to argue that some databases had a significant negative or positive impact on the outcomes. Thus, it can be concluded that mixing the datasets had no negative impact on the method’s performance.

### 5.2. Verification through Identification

As with the baseline methods, each of the five datasets contained a different share of separate databases in the training and testing datasets (Table 5).

To compare the baseline methods with the neural networks, the same models were selected for the VTI method, which was only trained using the ImageNet database. In the previous section, the training parameters of neural network models for the VTI method were described. The validation set contained 30% of the images from the testing set.

**Figure 5:**
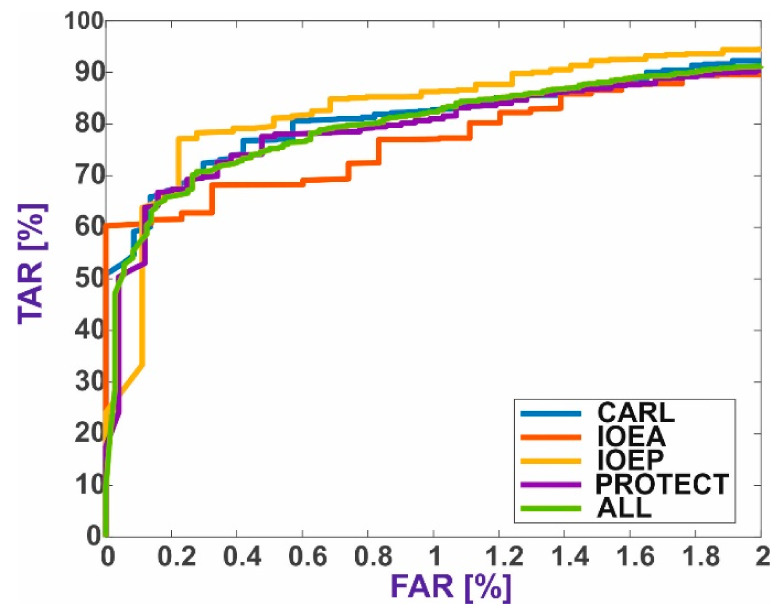
ROC curves for individual databases for the VTI method.

The best results for the VTI method were achieved using the Inception-v3 model (Table 6), due to a different data structure. The inception module for this type of data structure gave better results than simply extracting features from a single face image. The primary purpose of the inception module was to learn small details, medium-sized features, or almost entire images if they appear very often. It can be assumed that the inception module in accordance with its intended purpose extracts significantly more details for double-face images. Models with residual modules, even if based on inception modules, did not work well in the two-class classification task. To obtain these results, the neural network was trained by an average of 31 epochs. Neural network models with many parameters, such as VGG16 or AlexNet, also achieved low TAR values. These neural networks were trained by an average of 37 epochs. It was confirmed that CNNs with inception modules are trained faster than simple CNNs with many parameters, such as VGG16 or AlexNet. The DenseNet-201 model also had significantly fewer parameters and achieved high TAR values for FAR = 1%. However, to also achieve high values for FAR = 0.1%, extended structures of inception modules extracting smaller-sized features are needed. The inception modules from the first version of the Inception-v1 network (GoogLeNet) contained inception modules that extract fewer details. In Figure 5, we present the ROC curves for individual databases selected for the experiment. It can also be concluded that the mixing of databases did not have a negative impact on the TAR values.

A natural problem that arises immediately when analyzing the VTI method is whether it is sensitive to the order of images in the double image. Considering two component images, image 1 and image 2, they give two combinations of component images called “12” and “21”.

The results presented in Table 6 were developed for each training of a CNN model with a randomly mirrored data augmentation. The random augmentation means that not all images included in the training dataset are given in both combinations of component images. Identifying this information is crucial for further analysis, which involves checking what would happen if the amount of data increased not randomly, but exactly twice. 

For this purpose, we performed an additional training of the Inception-v3 model for data with a full augmentation instead of a random one. Table 7 shows the results of the experiment with the image positions swapped.

For FAR = 1%, TAR differences between combinations “12” and “21” are small in the case of both augmentations, while for FAR = 0.1%, the differences are greater and equal to about 10 percentage points for the random augmentation. It can be concluded that the full augmentation process did not have a high impact on the performance and can be omitted.

### 5.3. Results Summary

**Figure 6:**
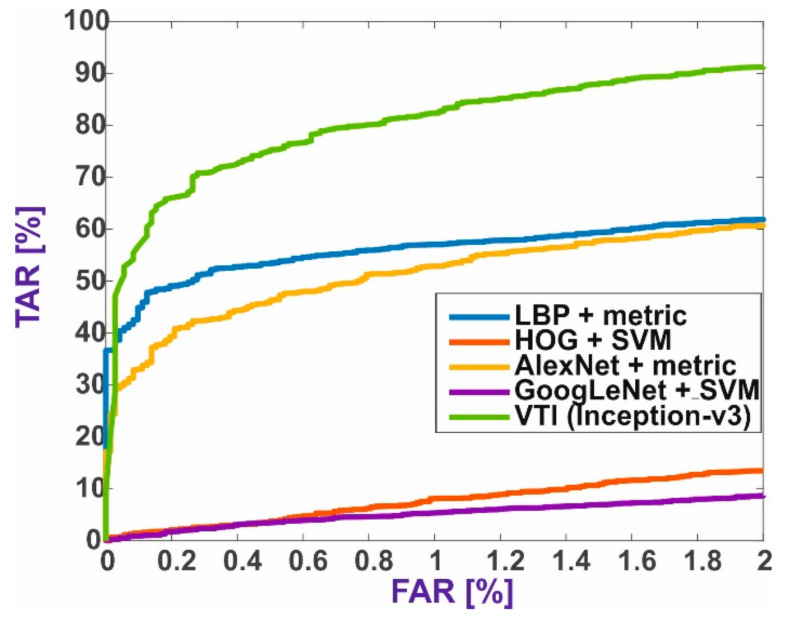
The best ROC curves for five face verification methods.

Figure 6 summarizes the results of all methods and shows the best ROC curves for four baseline and VTI methods.

The results prove that better results can be obtained for training with the decision function based on metrics than on the SVM function. The same data in the form of feature vectors were used in each experiment for each method. 

It cannot be generalized that neural networks are better at extracting features than local descriptors, but some of them, such as AlexNet, DenseNet-201, or ResNet-18, have better results for TAR than LDP or HOG algorithms. The algorithm for extracting LBP features is comparable with the mentioned CNNs, but it is possible that neural network models trained initially in the ImageNet extract general features from the image. If the models had been trained prior to the experiment on the same type of data, i.e., thermal images, it is possible that the convolutional layers would have detected more significant facial shapes and features.

The proposed VTI method achieves a TAR of 83.54% for the experiment with different datasets. This performance is better than that of the baseline methods described in this paper. The method proposed by Rodríguez-Pulecio et al. [2] achieved Rank-1 of 86.6% by using thermal signature templates with natural scene statistic features. However, their method is not fully automatic and requires manual landmark annotation before the actual data processing. In contrast, VTI is fully automatic and may be applied with any classifier.

## 6. Summary

In this paper, we present a new method for face verification based on a composition of two images and binary classification. The method was applied and validated for thermal infrared facial images, but could be potentially applied to other spectra as well. The proposed method draws inspiration from the Siamese architecture and identification process. However, it is not a typical identification because it considers only two classes. The method shows an improvement in comparison with other baseline methods based on local descriptors and CNNs. The presented results show that the order of combining pictures has no effect on the method’s performance.

The results obtained show that the longwave infrared face recognition can provide reliable performance. Since the proposed method is universal, further investigations with new architectures and new applications are planned. We plan to use the method for thermal-to-visible face recognition.

## Figures and Tables

**Table 1:** Parameters of the algorithms used in the study.

Name of Method	Parameters
Histogram of Oriented Gradients	cell size: 30 × 30 pixels number of cells in a block: 2 × 2 overlapping of a block: 1 × 1
Local Binary Pattern	cell size: 30 × 30 pixels number of neighboring pixels: 8 L2 normalization with a linear interpolation
Local Derivative Pattern	cell size: 30 × 30 pixels L2 normalization

**Table 2:** Percentage share of each database in the appropriate training and test dataset for local descriptor and CNNs models.

Training Datasets				
Number of Datasets	IOEA [%]	IOEP [%]	PROT [%]	CARL [%]
1	13.04	23.91	34.78	28.27
2	9.78	22.83	31.52	35.87
3	11.96	19.57	36.96	31.51
4	11.96	23.91	36.96	27.17
5	10.87	26.09	34.78	28.26
**Testing Datasets**				
**Number of Datasets**	**IOEA [%]**	**IOEP [%]**	**PROT [%]**	**CARL [%]**
1	10.00	17.50	35.00	37.50
2	17.50	20.00	42.50	20.00
3	12.50	27.50	30.00	30.00
4	12.50	17.50	30.00	40.00
5	15.00	12.50	35.00	37.50

**Table 3:** TAR results for the local descriptor and CNN models with the decision function based on metrics.

Local Descriptor Methods			
Algorithm of Feature Extraction	Distance Function	TAR @ FAR 1%	TAR @ FAR 0.1%
HOG	Spearman	48.46 ± 4.11	36.22 ± 4.50
LBP	Spearman	56.90 ± 3.13	40.33 ± 5.65
LDP	Spearman	35.25 ± 1.83	17.88 ± 2.51
**Models of CNNs**			
AlexNet	Spearman	53.36 ± 2.31	35.32 ± 3.83
DenseNet-201	Spearman	53.15 ± 4.67	34.13 ± 6.16
GoogLeNet	Euclidean	48.18 ± 2.51	26.85 ± 2.97
InceptionResNet-v2	Spearman	45.01 ± 4.86	26.49 ± 5.19
Inception-v3	Spearman	46.33 ± 3.97	29.71 ± 4.08
ResNet-18	Euclidean	53.60 ± 2.04	33.04 ± 2.49
ResNet-50	Euclidean	49.56 ± 4.01	29.92 ± 3.48
ResNet-101	Spearman	49.50 ± 4.73	30.73 ± 5.51
VGG16	Euclidean	44.08 ± 2.06	26.19 ± 2.55
VGG19	Euclidean	48.68 ± 3.27	28.69 ± 6.17

**Table 4:** TAR results for the local descriptor and CNN models with SVM.

Local Descriptors Methods		
Algorithm of Feature Extraction	TAR @ FAR 1%	TAR @ FAR 0.1%
HOG	8.94 ± 1.88	1.99 ± 1.34
LBP	5.08 ± 6.81	0.86 ± 1.21
LDP	5.95 ± 3.16	1.65 ± 1.19
**Models of CNNs**		
AlexNet	6.35 ± 3.12	0.36 ± 0.18
DenseNet-201	4.03 ± 0.72	0.10 ± 0.10
GoogLeNet	9.12 ± 2.02	1.44 ± 0.43
InceptionResNet-v2	2.72 ± 1.09	0.25 ± 0.25
Inception-v3	5.15 ± 2.96	0.32 ± 0.38
ResNet-18	6.42 ± 1.74	1.01 ± 0.55
ResNet-50	4.39 ± 2.45	0.32 ± 0.26
ResNet-101	4.90 ± 1.95	0.44 ± 0.92
VGG16	5.74 ± 3.27	0.99 ± 0.95
VGG19	5.85 ± 2.50	1.24 ± 1.15

**Table 5:** Percentage share of each database in the training and testing datasets for the VTI method.

Training Datasets				
Number of Datasets	IOEA [%]	IOEP [%]	PROT [%]	CARL [%]
1	11.96	20.65	34.78	32.61
2	13.04	21.74	36.96	28.26
3	10.87	20.65	35.87	32.61
4	10.87	26.09	34.78	28.26
5	15.22	20.65	36.96	27.17
**Testing Datasets**				
**Number of Datasets**	**IOEA [%]**	**IOEP [%]**	**PROT [%]**	**CARL [%]**
1	12.50	25.00	35.00	27.50
2	10.00	22.50	30.00	37.50
3	15.00	25.00	32.50	27.50
4	15.00	12.50	35.00	37.50
5	5.00	25.00	30.00	40.00

**Table 6:** TAR results for the VTI method.

Neural Network Model	TAR @ FAR 1%	TAR @ FAR 0.1%
AlexNet	61.57 ± 14.89	21.14 ± 10.09
DenseNet-201	78.16 ± 7.29	11.01 ± 24.63
GoogLeNet	58.00 ± 12.06	4.71 ± 10.53
InceptionResNet-v2	64.66 ± 9.81	9.76 ± 21.83
**Inception-v3**	**83.54 ± 7.04**	**60.22 ± 14.26**
ResNet-18	59.92 ± 9.06	20.51 ± 13.44
ResNet-50	57.89 ± 9.01	8.24 ± 11.46
ResNet-101	58.28 ± 10.23	15.42 ± 14.91
VGG16	63.64 ± 11.57	17.23 ± 15.91
VGG19	60.25 ± 6.76	19.82 ± 18.33

**Table 7:** Test results for different sample image and test image positions for the VTI method for the Inception-v3 model.

Full Augmentation		
Location of Images	TAR @ FAR 1%	TAR @ FAR 0.1%
“12”	83.92 ± 6.96	49.24 ± 19.08
“21”	85.33 ± 11.53	52.81 ± 15.69
**Random augmentation**		
“12”	83.54 ± 7.04	60.22 ± 14.26
“21”	83.39 ± 8.94	51.46 ± 13.04

## Data Availability

Not applicable.
